# Time‐course quantitative mapping of caffeine within the epidermis, using high‐contrast pump–probe stimulated Raman scattering microscopy

**DOI:** 10.1111/srt.13088

**Published:** 2021-10-07

**Authors:** Risa Iguchi, Yoji Nishi, Tsuyoshi Ogihara, Terumasa Ito, Fumiaki Matsuoka, Kazuhiko Misawa

**Affiliations:** ^1^ R&D Department Matsumoto Trading Co., Ltd. Tokyo Japan; ^2^ Department of Applied Physics Tokyo University of Agriculture and Technology Tokyo Japan

**Keywords:** label‐free imaging, real‐time imaging, skin penetration, stimulated Raman scattering microscopy

## Abstract

**Background:**

An assessment of the drug penetration and distribution profiles within the skin is essential in dermatology and cosmetology. Recent advances in label‐free imaging technologies have facilitated the direct detection of unlabeled compounds in tissues, with high resolution. However, it remains challenging to provide quantitative time‐course distribution maps of drugs within the complex skin tissue. The present study aims at acquiring the real‐time quantitative skin penetration profiles of topically applied caffeine, by means of a combination of pump**–**probe phase‐modulated stimulated Raman scattering (PM‐SRS) and confocal reflection microscopy. The recently developed PM‐SRS microscopy is a unique imaging tool that can minimize strong background signals through a pulse‐shaping technique, while providing high‐contrast images of small molecules in tissues.

**Materials and methods:**

Reconstructed human skin epidermis models were used in order to analyze caffeine penetration in tissues. The penetration profiles of caffeine in an aqueous solution, an oil‐in‐water gel, and a water‐in‐oil gel were examined by combining PM‐SRS and confocal reflection microscopy.

**Results:**

The characteristic Raman signal of caffeine was directly detected in the skin model using PM‐SRS. Integrating PM‐SRS and confocal reflection microscopy allowed real‐time concentration maps of caffeine to be obtained from formulation samples, within the skin model. Compared with the conventional Raman detection method, PM‐SRS lowered the background tissue‐oriented signals and supplied high‐contrast images of caffeine.

**Conclusion:**

We successfully established real‐time skin penetration profiles of caffeine from different formulations. PM‐SRS microscopy proved to be a powerful, non‐invasive, and real‐time depth‐profile imaging technique for use in quantitative studies of topically applied drugs.

## 1 INTRODUCTION

As the largest organ of the human body, the skin acts as a barrier against the environment. The outermost (upper) layer of the skin, the *stratum corneum* (*SC*), is composed of corneocytes and intercellular lipids, such as cholesterol, free fatty acids, and ceramides, and mainly provides a rate‐limiting barrier, which prevents not only excessive water loss from the body, but also the invasion of toxic chemicals and microbes.^1^, ^2^ Topically applied drugs, used in treating dermatological diseases, and active ingredients for cosmetic purposes, are examples of agents that need to cross the mentioned barrier to be effective inside the skin. Therefore, understanding the kinetics of both penetration and distribution of the applied drugs is crucial for pharmaceutical and cosmetic research.

While assessing the mechanism of skin penetration, time‐course quantitative maps of the target molecules are ideally required. There are several conventional methods for studying skin penetration, including the Franz diffusion cells and tape‐stripping. The Franz diffusion cell system combines ex vivo skin explants or in vitro reconstructed human skin cells, assembled on diffusion cells, with the appropriate quantification methods, such as liquid scintillation spectrometry for radiation‐labeled compounds^3^ or high‐performance liquid chromatography (HPLC).^4^, ^5^ Using these techniques, quantitative profiles can be obtained for topically applied compounds that reach the receiver chamber under the skin samples. Tape‐stripping, in turn, is a minimally invasive technique that can be applied in vivo. Briefly, it consists of removing the SC through the repeated application of adhesive tapes, followed by measurement of the amount of target molecules extracted from each tape, also giving rise to concentration profiles across the SC.^6^ Although these methods have been validated and widely applied, they are unable to depict with enough detail the time‐course distribution maps of compounds of interest in the skin layers.

Thus far, methodologies based on Raman spectroscopy have attracted attention in the field of skin research, because they are label‐free and non‐invasive processes. Raman spectroscopy has been employed to define skin penetration profiles of topically applied substances, both in vitro and in vivo,^7^, ^8^, ^9^, ^10^, ^11^, ^12^, ^13^, ^14^, ^15^ and in the analysis of the molecular composition of the skin samples.^16^ Nevertheless, confocal Raman microscopy^7^, ^8^, ^9^, ^10^, ^11^, ^12^, ^13^ does not support real‐time image acquisition because of the weak signal, whereas conventional stimulated Raman scattering (SRS) microscopy^14^, ^15^ suffers from low signal‐to‐background ratios because of the strong background signals from tissues. These obstacles limit real‐time imaging and signal detection especially at lower concentrations. In practice, a more sensitive and highly specific detection technique is needed to better understand the precise penetration kinetics of molecules of interest.

With our current work, we aimed to acquire penetration profiles of caffeine, which is widely used as a reference for skin penetration studies, in dermatology and also cosmetology,^4^, ^8^, ^9^, ^10^ released from different types of formulations ‐ more exactly, an aqueous solution, a water‐in‐oil gel, and an oil‐in‐water gel. We opted for the use of the novel pump–probe phase‐modulated (PM) SRS microscopy, which minimizes strong background signals with a pulse‐shaping technique, thereby producing high‐contrast imaging of small molecules in the tissues of interest.^17^, ^18^ As presently established, the manner in which active molecules penetrate the skin depends not only on their own physical and chemical properties, but also on the characteristics of their delivery vehicle. Thus, the formulation samples of a disparate nature were applied to the artificial epidermis models. Ultimately, this recent microscopy technology has made direct in vitro visualization of time‐dependent depth profiles of caffeine in skin models, using different formulations as vehicles.

## 2 MATERIALS AND METHODS

### 2.1 Substances and formulations

Caffeine (Shizuoka Coffein Co., Ltd., Shizuoka, Japan) was utilized as the unlabeled model target for microscopic visualization. For the aqueous solution sample, caffeine was dissolved in pure water, at room temperature and 1% w/w. Regarding the water‐in‐oil and oil‐in‐water gel formulations, they each contained 1% w/w caffeine.

#### 2.1.1 Water‐in‐oil gel

We prepared a silicone‐based water‐in‐oil gel with 0.2% w/w silicone‐based emulsifiers (PEG‐9 polydimethylsiloxyethyl dimethicone and polyglyceryl‐3 polydimethylsiloxyethyl dimethicone), 0.4% w/w silicone elastomers (dimethicone/PEG‐10/15 crosspolymer and dimethicone/vinyl dimethicone crosspolymer), 6% w/w silicone oils (dimethicone and cyclopentasiloxane), 40% w/w glycerine, 5% w/w ethanol, 1% w/w sodium chloride, 0.5% w/w phenoxyethanol, 45.9% w/w pure water, and 1% w/w caffeine.

#### 2.1.2 Oil‐in‐water gel

The composition of the oil‐in‐water gel was as follows: 0.4% w/w ammonium acryloyldimethyltaurate/beheneth‐25 methacrylate crosspolymer, 38% w/w glycerine, 1% w/w squalane, 0.4% w/w phenoxyethanol, 59.2% w/w pure water, and 1% w/w caffeine. Caffeine was first dissolved in pure water. The gels were then prepared by premixing the oil‐in‐water and hydrophobic phases. Next, the oil‐in‐water and hydrophobic components were mixed by a homo mixer, at 5000 rpm for 2 min.

### 2.2 Sample preparation for microscopic imaging

Reconstructed human skin epidermis models (EPI‐606X; MatTek, Ashland, MA) were used for the analysis of caffeine skin penetration. After cutting the skin from the cell culture insert and removing the cell culture substrate, the skin model (20 mm in diameter) was assembled on a glass slide with a single cavity. The space between the lower, basal cell layer of the tissue and the surface of the glass slide cavity was filled with the receiver solution: 20 μL of phosphate‐buffered saline. For the study of aqueous solution samples, a 1 mm‐thick steel washer was placed on each skin model and 50 μL of an aqueous caffeine solution was dropped directly onto the surface, within the inner hole of the washer. Finally, a coverslip was placed on top of the washer, in order to enclose the solution. The interfaces between the washer and the skin model and the washer and the coverslip were sealed with silicone grease.

For the caffeine gels, a 0.06 mm‐thick adhesive tape with a hole was applied, instead of the 1 mm‐thick steel washer, to minimize the optical scattering loss, owing to the heterogeneity of the formulations. The rest of the conditions were equal to those of the solution samples.

Finally, all samples were placed on the stage of the PM‐SRS microscope and images were recorded as described below.

### 2.3 PM‐SRS imaging

**FIGURE 1:**
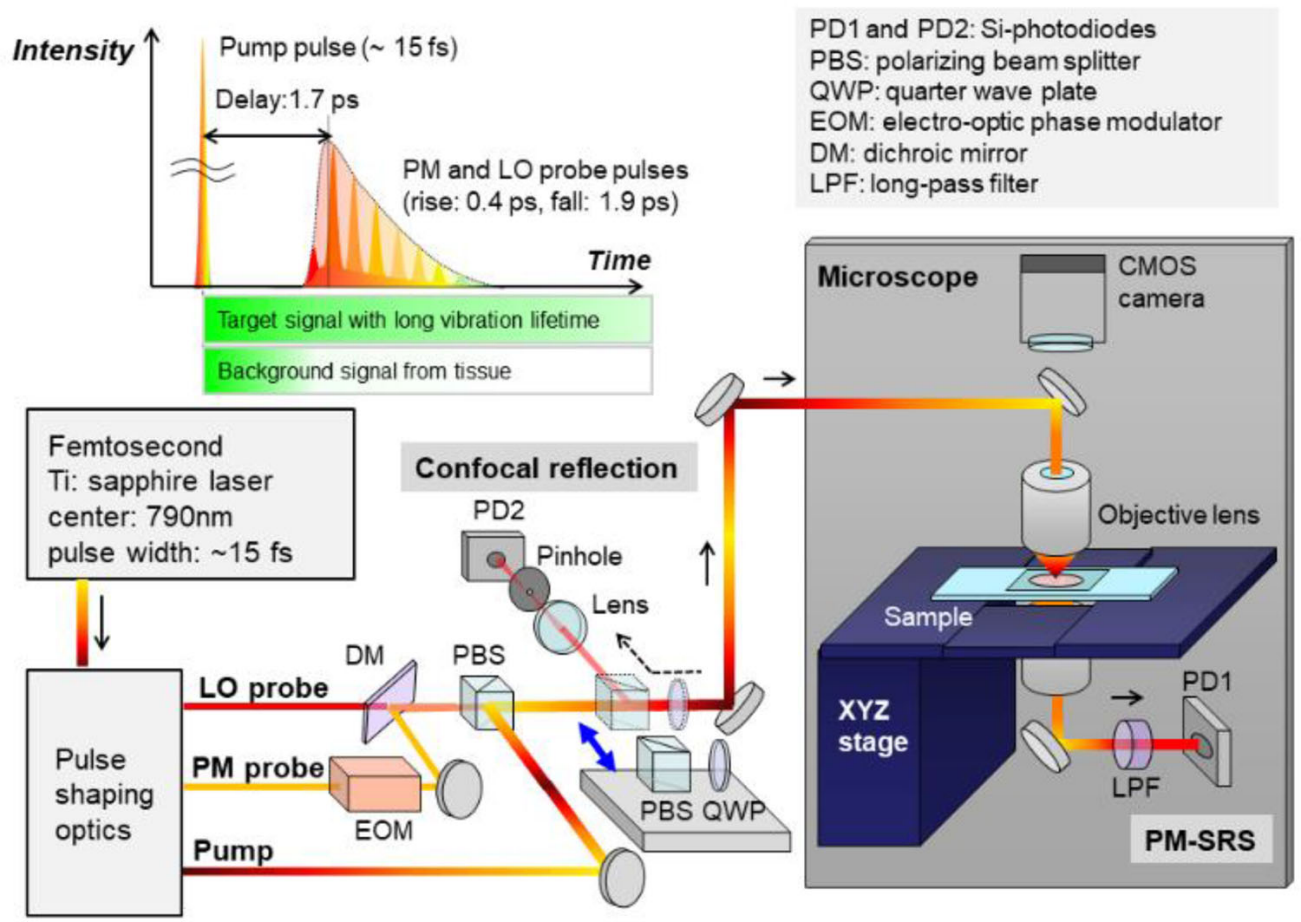
Schematic of the combined PM‐SRS and confocal reflection microscope. In the PM‐SRS mode, a femtosecond excitation pulse (the pump pulse) and two probe pulses (the PM and the LO probe pulses with a 1.7 ps time delay) are combined and focused onto the sample. The SRS signal on the transmitted LO light is detected by PD1, followed by demodulation using a lock‐in amplifier. In the confocal reflection mode, the PBS and QWP are inserted, and the reflected light are detected through PD2

The setup of our PM‐SRS microscope was designed to allow switching between the PM‐SRS mode, for tracing the Raman signals of the target molecules, and the confocal reflection mode, for acquiring morphological information of the tissue sample. The principles of PM‐SRS and the detailed setup of the PM‐SRS microscope in this study were previously described.^17^, ^18^ Figure 1 shows a schematic of the PM‐SRS microscope. A femtosecond pulse generated by a Ti‐sapphire laser (Vitara, Coherent: 80 MHz, 15 fs, and centered at 790 nm) was split into pump and probe pulses, the latter of which was further divided into two probe pulses. A femtosecond pump pulse initiates the coherent vibration of all molecules in the focal volume, and the specific target Raman mode is selectively detected by adjusting the difference in frequencies between the two probe pulses. To achieve a superior signal‐to‐background ratio, the two probe pulses are both delayed with respect to the pump pulse by 1.7 ps, and then stretched and shaped to achieve a pair of time‐asymmetric picosecond pulses with distinct center frequencies. The optimized pulse shaping enables us to efficiently reject background signals from tissue Raman scattering and other nonlinear optical responses, and selectively detect the long‐lived vibrational signal of caffeine. For SRS signal detection, one of the probe pulses (the PM probe) is modulated by an electro‐optic modulator (EO‐PM‐NR‐C1, Thorlabs), whereas the non‐modulated probe is defined as a local oscillator (LO) probe pulse. All laser pulses were combined (for a total laser power of 50 mW) and focused onto the sample with an objective lens (M Plan Apo NIR 100x, NA 0.50, Mitutoyo). The heterodyne SRS output signal on the transmission LO probe pulse, which is proportional to the concentration of the target molecule, is then detected using an Si photodetector (PDA36A, Thorlabs) and also demodulated with a lock‐in amplifier (LI5640, NF Corporation). For the confocal reflection microscopy mode, back‐scattered light was collected by the objective lens, and the intensity was measured by another identical Si photodetector. Two‐dimensional depth‐resolved images were recorded through XZ raster scanning, by driving the sample stage. The data acquisition period for each XZ SRS image was 100 s.

## 3 RESULTS

### 3.1 Raman spectra of skin model and caffeine

**FIGURE 2:**
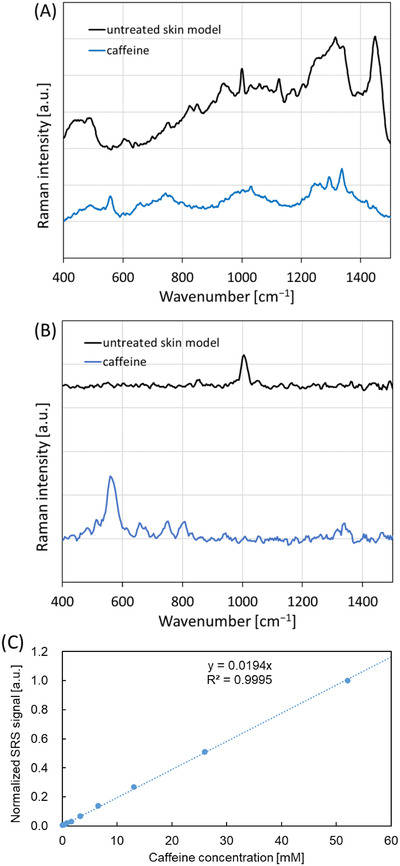
(A) Confocal Raman spectra and (B) PM‐SRS spectra of untreated skin model, close to the surface, and of a 1% caffeine solution. (C) Caffeine calibration curve obtained using PM‐SRS microscopy at 560 cm^−1^, with the appropriate linear fit (mean ± 2SE, n = 100)

Prior to the penetration study, an untreated skin model sample and caffeine solution were analyzed using a self‐made confocal Raman spectroscope with a 532 nm CW excitation laser and a PM‐SRS microscope within the 400−1500 cm^−1^ range. The spontaneous Raman spectrum close to the surface of the untreated skin model showed some specific bands generated from the skin model components (Figure 2A). More precisely, the ring‐breathing mode in aromatic amino acids, shown at 1004 cm^−1^, the CH_2_ deformation mode generated from alkyl chains in lipids, at 1314 cm^−1^, and an unspecific C‐H deformation, present at 1448 cm^−1^,^19^ were quite noticeable. By contrast, the spectrum resulting from the PM‐SRS measurement, with a 1.7 ps probe delay, suppressed these background signals from the skin model components (Figure 2B), apart from the one corresponding to the ring‐breathing mode, with the origin in the aromatic amino acids as mentioned above, for 1004 cm^−1^. A characteristic signal from caffeine was detected at 560 cm^−1^ (vibration from the O=C‐N deformation^20^), similar to the confocal Raman and PM‐SRS spectra. Notwithstanding, the signal‐to‐background ratio of the PM‐SRS spectrum was significantly higher than that of the confocal Raman spectrum, given the long lifetime of the caffeine‐specific vibration.

Figure 2C shows the calibration curve of the caffeine solution, obtained using PM‐SRS at 560 cm^−1^. A linear fit is shown for the concentration range of 0 to 52.1 mM.

### 3.2 Skin penetration profile of caffeine

**FIGURE 3:**
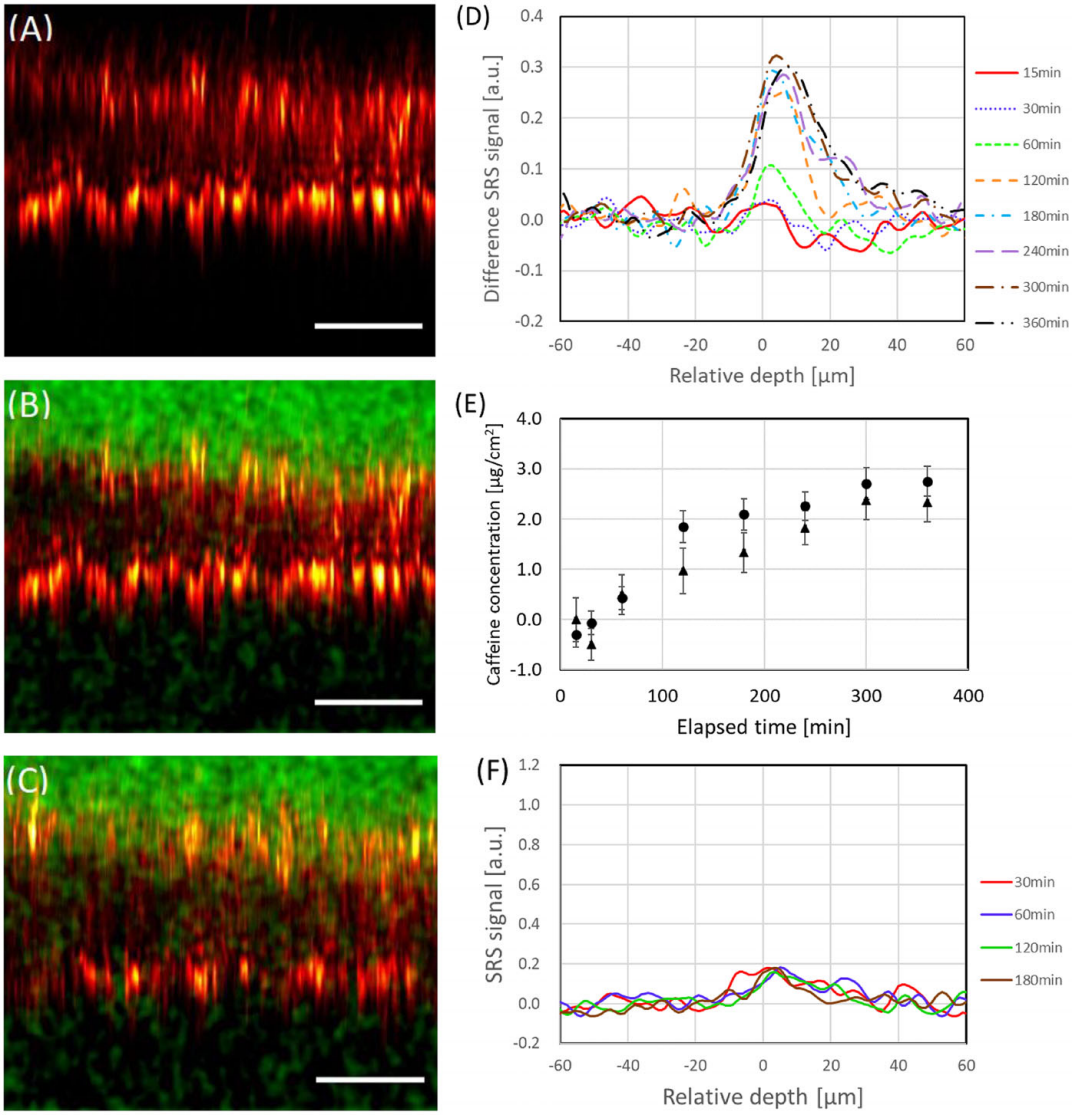
(A) Confocal reflection image of the XZ section of a skin model 15 min after treatment with 1% caffeine solution. Overlapped images of confocal reflection (red) and PM‐SRS signal at 560 cm^−1^ (green) of a skin model (B) 15 min after or (C) 360 min after being treated with 1% caffeine solution. PM‐SRS signals were accumulated twice. (D) Real‐time depth profiles of caffeine (560 cm^−1^) in the skin model (15−360 min after applying the drug), where the baseline SRS signal of the aqueous caffeine solution is subtracted. (E) Concentration profile of caffeine over time, within a depth of 5−15 μm from the relative zero‐depth (n = 2; dots and triangles). Error bars represent twice the standard error based on the system noise. (F) Control real‐time profiles at 560 cm^−1^ in the skin model (30−180 min). Scale bar = 50 μm

The confocal reflection XZ image of this model was acquired 15 min after applying 1% caffeine solution (Figure 3A). Strong signals were observed at both the upper and lower depth positions. By comparing the confocal signals and the Raman signal at 1004 cm^−1^, which originated from the aromatic amino acids of the *SC*, we found that the strong reflection signals at the top were generated from the middle of the *SC* and defined the relative zero‐depth of the skin model as the position where the confocal reflection signal is at maximum in each study. The confocal reflection peak perceived at the bottom was from the interface between the basal cell layer and the buffer solution, owing to the elevated refractive‐index mismatch. Such a contrast can be used to monitor the local morphology and partly unveil the layer structure of the skin model. Based on the confocal signal peaks presented, the tissue thickness was estimated to be approximately 50 μm.

Furthermore, the overlaid XZ images of the confocal reflection (red signal) and PM‐SRS microscopy at 560 cm^−1^ (green signal), attained at 15 and 360 min after applying the 1% caffeine solution, are shown in Figures 3B and 3C, respectively. These images correspond to the high‐contrast two‐dimensional distribution maps of topically applied caffeine. Whereas the PM‐SRS signal of caffeine at 560 cm^−1^, with regard to the solution sample, was detected only above the upper surface at 15 min, a fraction of the signal was also noticed within the skin model 360 min after the drug was added. To quantitively analyze the skin penetration, we calculated the depth profile of the caffeine signal from the PM‐SRS imaging data. Here, the relative zero‐depth position was defined by the selection of a confocal signal peak along the *Z*‐direction, and the SRS signal intensity was normalized with the corresponding signal level measured for the 1% caffeine solution. In addition, Figure 3D represents the difference in the SRS signal profile (the original depth profile–the baseline depth profile), where the baseline is established through the convolution of a step function (1 for Z < 0 and 0 for Z > 0) and the instrument response function (Lorentz function). By examining the real‐time depth‐profile data, which integrated the values of the SRS signal at 560 cm^−1^, within a depth of 5−15 μm from the relative zero‐depth, these were converted into concentration levels relative to the caffeine calibration curve (Figure 3E), revealing that such molecules penetrated the skin model in a time‐dependent manner, saturating the tissue after 180 min. Still encompassed in the aqueous solution assay, Figure 3F shows the control depth profiles of the normalized signal at 560 cm^−1^, corresponding to the case of no caffeine (30−180 min), substantiating the idea that the tissue background signal was suppressed by the caffeine signal levels with concentrations of above 0.1%. The detection limit of caffeine in the skin model employed here was estimated to be approximately 0.1% (5.2 mM), based on the results above.

### 3.3 Distribution maps of formulation samples

**FIGURE 4:**
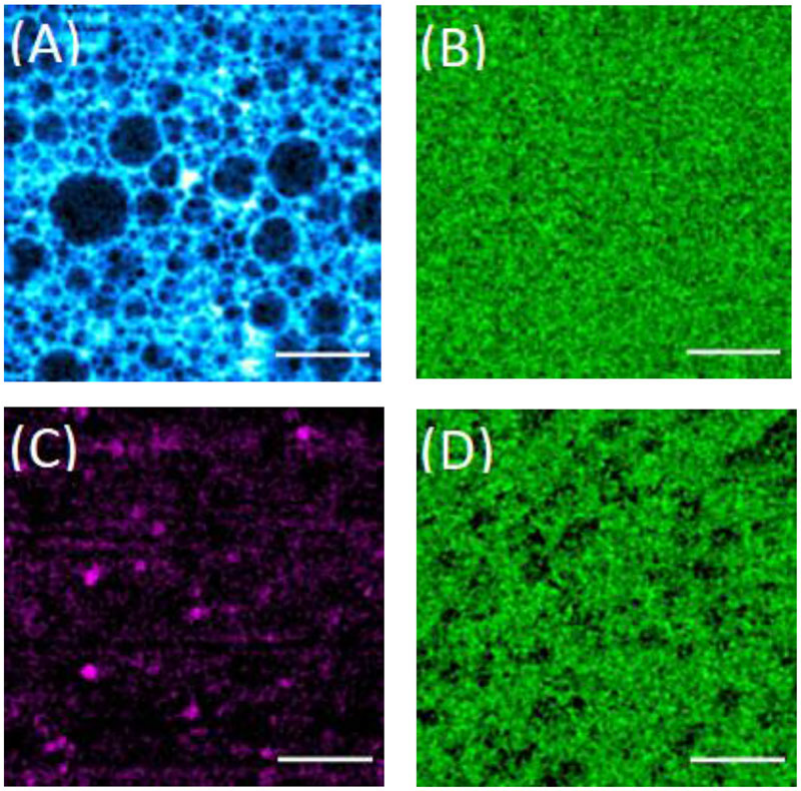
Distribution maps of (A) silicone oil (480 cm^−1^) and (B) caffeine (560 cm^−1^) in a water‐in‐oil gel, and of (C) squalane (1440 cm^−1^) and (D) caffeine (560 cm^−1^) in an oil‐in‐water gel. Scale bar = 50 μm

The formulation samples were directly analyzed using PM‐SRS, among which Figures 4A and 4B are the distribution maps of silicone oil (480 cm^−1^) and caffeine (560 cm^−1^) obtained with the water‐in‐oil gel. Figures 4C and 4D, in turn, portray those of squalane (1440 cm^−1^) and caffeine (560 cm^−1^) and were captured using the oil‐in‐water gel. In terms of the distribution maps of the formulation samples, our study suggests that caffeine density was superior in the water phase of the oil‐in‐water gel. By contrast, caffeine was more evenly distributed in the water and oil phases in the water‐in‐oil gel, which may be due to the fact that caffeine is soluble in both water and ethanol, and ethanol is also soluble in the low‐viscosity dimethicone used for the gel.

### 3.4 Skin penetration profiles of caffeine from formulation samples

**FIGURE 5:**
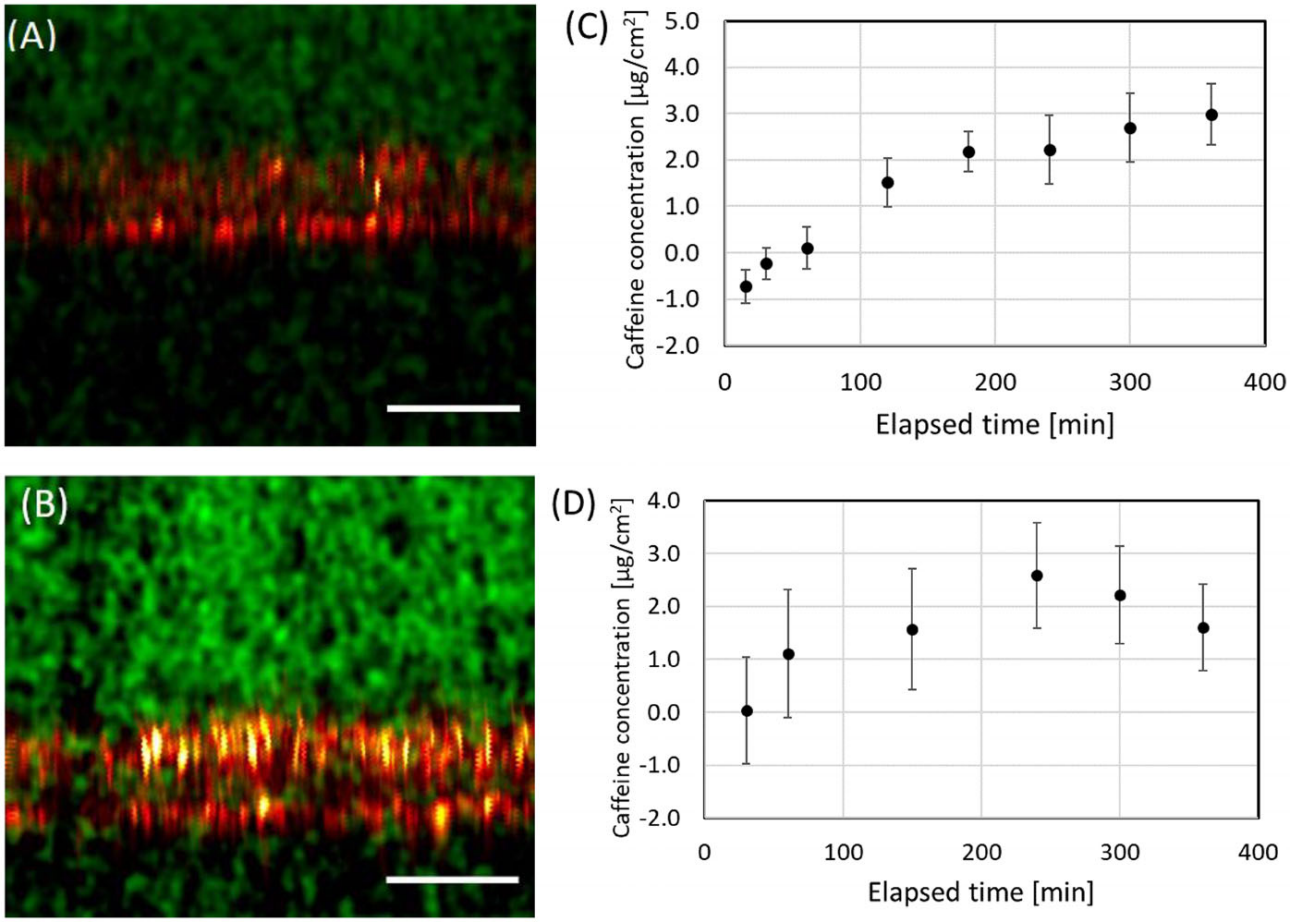
Overlapped images of confocal reflection (red) and PM‐SRS signal at 560 cm^−1^ (green) of skin models, (A) 360 min after treatment with the 1% caffeine water‐in‐oil gel and (B) 360 min after applying 1% caffeine oil‐in‐water gel. PM‐SRS signals were accumulated twice for the water‐in‐oil gel and eight times for the oil‐in‐water gel. Concentration profiles of caffeine, over time, from (C) the water‐in‐oil gel and of (D) the oil‐in‐water gel, within a depth of 5−15 μm from the relative zero‐depth. Error bars represent twice the standard error based on the system noise. Scale bar = 50 μm

Finally, Figure 5A depicts the overlapped images of the PM‐SRS and the confocal reflection modes produced 360 min after applying 1% caffeine water‐in‐oil gel on the skin model. The equivalent overlapped image for the oil‐in‐water formulation is shown in Figure 5B, 360 min after applying 1% caffeine oil‐in‐water gel to the tissue models. Images of the PM‐SRS signal at 560 cm^−1^ obtained over time were used to calculate the concentration profiles of caffeine, once again within a depth of 5−15 μm from the relative zero‐depth, of the water‐in‐oil gel and the oil‐in‐water gel application, using the calibration curves (Figures 5C and 5D). These results support the idea that caffeine clearly penetrated the skin models over time from both the water‐in‐oil and oil‐in‐water gel formulations.

## 4 DISCUSSION

In the process of confocal Raman analysis of biological tissues, Raman signal attenuation according to depth is one of the core challenges. Since the cosmetic formulation samples usually consist of numerous ingredients and are not always homogeneous, the refractive‐index difference of the samples tends to become more extensive in comparison with pure solution samples. As a result, strong scattering from the formulation samples significantly affects the optical transmission of the laser, thus disturbing the measurement of the Raman signals from the target compound. The optical scattering loss could be even more problematic in conventional confocal Raman microscopy, particularly when dealing with a 532 nm green laser setup. Despite that, the PM‐SRS method could overcome this limitation and provide penetration profiles for both oil‐in‐water and water‐in‐oil samples, because of its higher sensitivity and reduced scattering, due to working with near‐infrared laser wavelengths. In the present study, we observed that the oil‐in‐water gel gave rise to stronger scattering and signal loss than the water‐in‐oil gel. The relative signal intensity, in this sense, was 0.64 for the water‐in‐oil gel and 0.17 for the oil‐in‐water gel, correspondingly, compared to 1.00 for the caffeine solution. Furthermore, PM‐SRS images of the water‐in‐oil (Figure 5A) and the oil‐in‐water (Figure 5B) gels were obtained twice and eight times accumulation, respectively.

When tracing the skin penetration profiles of certain molecules, PM‐SRS microscopy has another advantage compared with other Raman microscopy approaches. Confocal Raman microscopy and conventional amplitude‐modulated SRS microscopy detect strong tissue‐oriented Raman scattering, caused by components such as proteins, lipids, and water. Consecutively, high background hinders the detection of target molecules within tissues, and more so at lower concentrations. To overcome this drawback, several approaches have been proposed, including an analysis of the correlation data between the tissue‐oriented signal and the signal from a molecule of interest,^10^ as well as the use of a non‐negative matrix algorithm of depth‐resolved Raman spectra of the tissue.^13^ By contrast, PM‐SRS provides direct quantitative depth profiles of the target molecules, given that PM‐SRS can suppress the background signals from the skin samples and trace the characteristic Raman peaks from target substances, even at lower concentrations.

However, it is impossible to determine the actual surface location of the sample or the absolute skin depth profile from the data obtained from our setup alone. Therefore, the relative zero‐depth needs to be defined to discuss the depth profile. This is a limitation of the present study. For a more accurate discussion of the penetration depth, a higher depth resolution, a separation of the tissue components through multi‐color Raman spectral imaging, and an estimation from the depth‐specific histomorphological information will be needed. The use of multi‐color imaging is also expected to further improve the separation performance of skin components and skin‐like substances.

## 5 CONCLUSION

In this study, we demonstrated a label‐free microscopy‐based method to visualize the distribution maps of topically applied caffeine by means of an artificial skin model. The recent PM‐SRS technique supplied high‐contrast images of our target molecule within the skin model, after selectively probing the long‐lived Raman signal from the caffeine and rejecting the background signals. Taking advantage of such a combined approach of confocal reflection and PM‐SRS microscopy, we successfully imaged real‐time skin penetration of caffeine, released from different types of formulations. Interestingly, we noticed a disparate degree of optical scattering loss among the formulations. The small sample size indicates that further experimental work is required to discuss the detailed and precise skin penetration profiles of caffeine. Nevertheless, the present study sufficiently demonstrates the usefulness of PM‐SRS as a non‐invasive method to quantitatively evaluate the skin penetration of pure molecules as well as complex formulation samples in vitro.

## AUTHOR CONTRIBUTIONS

R.I., Y.N., T.I., and F.M. performed the research. R.I., T.O., T.I., and K.M. designed the research study. Y.N., T.I., and F.M. contributed essential reagents or tools. T.I. and F.M. analyzed the data. R.I. and T.I. wrote the paper.
